# Adolescents' Understanding of What Causes Emotional Distress: A Qualitative Exploration in a Non-clinical Sample Using Ideal-Type Analysis

**DOI:** 10.3389/fpubh.2021.673321

**Published:** 2021-05-24

**Authors:** Alisha O'Neill, Emily Stapley, Sarah Stock, Hannah Merrick, Neil Humphrey

**Affiliations:** ^1^Department of Education, The University of Manchester, Manchester, United Kingdom; ^2^Evidence Based Practice Unit (Anna Freud National Centre for Children and Families and University College London), London, United Kingdom

**Keywords:** emotional distress, adolescence, perceived cause, causal attribution, help-seeking, non-clinical, at risk adolescents

## Abstract

**Background:** There is increased interest in early intervention and prevention of mental health difficulties during adolescence; thus, we are seeing increased efforts to optimize well-being during this epoch. Positive emotional experiences are a central component of overall well-being. However, research exploring what adolescents perceive to be the cause(s) of their emotional difficulties is lacking. Improving understanding of this issue within non-clinical adolescent groups may provide useful insight into how to develop strategies to support young people as they navigate emotional difficulties.

**Objectives:** The aim of this research was to explore if meaningful categories of perceived cause(s) for emotional distress exist for non-clinical adolescent groups.

**Methods:** The data for this study were drawn from interviews across 6 sites in England conducted as part of the 5-year national evaluation of the HeadStart Learning Programme. The sample comprised of 32 young people aged 11–12 years from the first annual wave of qualitative data collection in 2017. Ideal type analysis—a qualitative form of person-centered analysis—was used to construct a typology of adolescents perceived cause(s) for emotional distress.

**Findings:** We identified five distinct categories of perceived cause: (1) *perceived lack of control*; (2) *unfair treatment*; (3) *others, their actions and judgements as the catalyst*; (4) *concerns for self and others; and*, (5) *self as cause*.

**Conclusions:** Our findings illustrate that distinct categories for perceived cause of emotional distress exist among adolescents considered to be “at risk” of developing mental health difficulties, which provides a foundation for future necessary work seeking to investigate the possible link between perceived cause for emotional distress and help-seeking behavior among sub-clinical groups.

## Introduction

Half of all lifetime mental health conditions begin before the age of 14 (1) and three quarters by the age of 18 (2). Accordingly, there is significant interest in adolescent well-being and the fortification of adaptive functioning to prevent the onset of mental health difficulties (3, 4). Poor mental health during adolescence is associated with a host of negative outcomes, including greater suicide risk (5) and increased risk of mental health concerns later in life (6). Unfavorable outcomes, as well as the significant economic “disease burden” of mental health difficulties (1, 7), illustrate the importance of early identification and prevention (8).

Yet, recent reports indicate that the prevalence of mental health problems among adolescents appear to be significantly higher than previously thought (9); indeed, probable mental health disorders during adolescence are rising [e.g., (10, 11)]. Health and Social Care Information Centre (11), for instance, found a 12.6% increase in probable mental health disorders from 2017 to 2020 in young people aged 11–16 years. There remains debate over whether this increase is due to improved recognition and referrals, or actual deterioration (5). For instance, shifts to broader definitions of mental health disorders and changing thresholds for clinical diagnosis could be contributing to the rise in prevalence (12) Nonetheless, numerous other factors may contribute to an overall increase, including the widespread use of social media (e.g., cyber bullying and engendering a greater need for validation); greater academic pressure due to increased governmental focus on high-stakes testing; and, lower family income as a result of recession (13). In addition, the COVID-19 pandemic and its consequences (e.g., lockdowns) may pose further risks to adolescent well-being (11). These factors may exacerbate what is already considered to be a turbulent developmental period.

Adolescence is typically understood as a challenging epoch owing to significant bio-social changes unique to this period (14), which may exacerbate stress levels and impact overall well-being (15). Notably, increased stress and subsequent stress appraisal can engender emotional distress—sometimes referred to as emotional problems, emotional symptoms, or emotional difficulties—should individuals feel ill-equipped to manage their difficulties (16, 17). Emotional distress refers to a heterogeneous collection of negative affect responses such as anger, depression, anxiety and sadness (16). Most young people will experience difficult emotions to some degree during adolescence (14). Emotions have important functions (18), therefore, difficult emotions do not need to be eradicated, but may require management to prevent the onset of mental health difficulties. Indeed, it is recognized that emotional distress may manifest into more serious disorders if not managed correctly, it can also stifle optimal well-being [e.g., (19)], which is an important outcome in its own right.

It should be noted that, the absence of psychopathology does not necessarily indicate wellness (20); therefore, adolescents who do not experience clinical levels of mental ill health may still experience suboptimal levels of well-being (21), which can be burdensome in terms of quality of life. According to Seligman (22), positive emotional experiences are a central component to overall well-being, thus emphasizing the importance of supporting young people who are experiencing emotional distress, not just for prevention, but also to bolster well-being. Nevertheless, unlike behavioral difficulties, emotional difficulties are less easily identifiable (23), placing a greater responsibility on young people to seek support when struggling. Taken together, these points highlight the value of improving non-clinical adolescents' adaptive help-seeking for emotional distress. However, whilst it is likely that a quarter of adolescents will encounter emotional and psychological difficulties, a mere third of those who do will seek help (23).

Encouraging young people to seek help when facing emotional difficulties is an important aspect of prevention (8), as adaptive help-seeking is a key facilitator to accessing appropriate support (24). The World Health Organization (25), defines adolescent help-seeking as the following:

*Any action or activity carried out by an adolescent who perceives herself/himself as needing personal, psychological, affective assistance or health and social services, with the purpose of meeting this need in a positive way*.

Thus, help-seeking is much broader and more complex than accessing therapeutic intervention through formal mental health networks. However, adolescents, more so than any other age group, are typically poor help-seekers when it comes to their mental health and emotional well-being (8, 26), and low help-seeking among adolescents remains a significant barrier to timely and effective intervention (8, 27). In a recent systematic review, Radez et al. (8) identified 4 key barriers to seeking professional help for children and adolescents, which included the following: (1) Individual factors, including limited mental health knowledge and perceptions of help-seeking; (2) Social factors, such as stigma and embarrassment; (3) Perceptions of therapeutic relationships, including considerations around trust and confidentiality; (4) Systemic and structural factors, including costs, logistics, and availability of help. The review by Radez et al. (8) focuses on professional help for (possible) clinical disorders as non-clinical samples are comparatively underexplored in help-seeking research. However, the findings do illustrate that help-seeking is a multifaceted process influenced by numerous internal and external factors. Nevertheless, despite a growing body of research investigating barriers and facilitators, poor help-seeking remains problematic within this age group. It is therefore clear that further exploration of the factors that may influence help-seeking behavior is pertinent.

According to the self-regulation model—commonly used to predict physical as well as mental illness behavior—causal beliefs are central to illness representation (27, 28). Indeed, it is well-recognized that perceived cause influences treatment and engagement preferences for physical illnesses (29, 30). Accordingly, there is a growing body of literature seeking to explore causal perceptions for mental health difficulties, and their subsequent influence on help-seeking [e.g., 29–32], in order to determine the ways in which perceived cause may be linked to help-seeking behavior. Empirical evidence has demonstrated that adults' views regarding the cause of their mental health difficulties are linked to subsequent help-seeking behavior. For instance, early research by Goldstein and Rosselli (31) found that endorsement of biological cause of depression led to a preference for psychotherapy, whereas the endorsement of a psychological model led to a preference to self-help, later work by Nieuwsma and Pepper (32) reported similar findings. More recent research, by Houle et al. (33), however, found that social causes were more likely to lead to a preference for psychotherapy; whereas, Stolzenburg et al. (28) found that biomedical attributions for depression appeared to be the only causal factors related to increase perceived need and intention to seek help. These findings may infer that person-related cause is linked to feelings of personal responsibility to manage difficulties, whereas bio-medical explanations may be associated with less personal responsibility (28, 34), possibly making individuals more likely to seek help. Though somewhat unequivocal, these findings demonstrate that aetiological beliefs influence help-seeking behavior in adulthood. However, much of the current research tends to lean toward statistical methods and the use of surveys [e.g., (26, 31–33)].

An understanding of mental illness and its origin is based on personal experiences and insights relevant to the individual (30); thus, perceptions of cause are inextricably linked to individual experiences. Therefore, qualitative research investigating perceived cause and help-seeking may prove pragmatic, as it seeks to illustrate participants' life-worlds, thereby contributing to a more holistic understanding of social realities and patterns of meaning (35). Indeed, whilst limited, research using qualitative methods to determine links between etiology and support seeking have provided promising insights. For instance, Nunstedt et al., (30) interviewed clinically depressed adults, finding that participants' aetiological beliefs influenced whether they sought treatment. The authors found that, for some participants, a lack of understanding of what was happening and what was causing their depression lead to a lack of understanding that they could seek help. The knowledge that a lack of understanding of cause may influence help-seeking is an important finding—illustrating further that causal perceptions are salient to help-seeking outcomes. However, despite the fact that the majority of mental health disorders begin during adolescence (1, 2), much of the research in this area remains focused on adulthood.

Considering the findings with adult groups, it is likely that perceived cause for emotional difficulties may also play a key role in adolescents' decisions to seek-help (29). Understanding and attributing cause involves complex cognitive processes (30, 36); therefore, as adolescents are still developing cognitively (36, 37) there are likely differences in the ways adults and adolescents attribute cause. Accordingly, it is necessary to pay adolescents specific research attention to understand how cause and help-seeking may be linked during this epoch. In an attempt to address the gap in adolescent research, Midgley et al. (29) investigated causal perceptions of adolescents with a clinical diagnosis of depression, finding three distinct themes: (1) bewilderment about why they were depressed; (2) depression as a result of rejection, victimization, and stress; and (3) something inside is to blame. Therefore, it is likely that adolescents do engage in causal thinking regarding their difficulties, but more work is needed to determine if causal attributions influence help-seeking. Furthermore, the focus of the work by Midgley et al. (29) was perceived cause of depression, overlooking non-clinical groups of adolescents who still experience emotional distress. Indeed, much of the work in this field focuses specifically on depression [e.g., (29–33, 38)].

Understanding the etiology of depression is an important research endeavor (39), however, it negates insight into how perceptions of cause might influence help-seeking for emotional difficulties in non-clinical groups who still experience sub-optimal levels of well-being, which may have important implications for the prevention of affective disorders and improving well-being more generally. Thus, understanding the interplay between cause and help-seeking in non-clinical groups is a valuable, yet under explored, research endeavor. In order to understand the extent to which perceived cause might influence help-seeking tendencies during adolescence, however, it is first necessary to explore whether or not meaningful categories for perceived cause exist for non-clinical groups, as this information is currently lacking.

As far as the authors are aware, no study has hitherto explored perceived cause for emotional distress with a group of participants considered to be “at risk” of developing mental health difficulties. Such investigation is pertinent as research with sub-clinical groups may further offer insight into how and when to effectively intervene to prevent difficulties from manifesting, as well as improve overall well-being; further, it may also help to improve the development of appropriate mental health interventions. The present study aims to address current gaps using ideal type analysis. Although perceptions of cause for depression during adolescence have previously been successfully explored using thematic analysis, ideal type analysis offers comparative analysis within and between types (40). Thus, as perceived cause is multifaceted and complex (30), ideal type analysis allows for the development of distinct categories of causal perception whilst also allowing for recognition and discussion of the complexities within and between types of cause. This paper is the first of a two-part series, starting with perceptions of cause for emotional distress. The second paper will build on the findings presented here, to explore if categories of perceived cause for emotional distress influence help-seeking among sub-clinical adolescent groups.

## Method

### Setting for the Study

The data for this study were drawn from interviews conducted as part of the national evaluation of the HeadStart Learning Programme. The HeadStart Learning Programme is a five-year programme which aims to improve the mental health and well-being of 10–16 year olds and prevent the manifestation of serious mental health issues. This is achieved through the implementation of a wide range of preventive interventions at six sites across England. It should be noted that this research was conducted in a Western setting, as research shows that explanatory models of mental health and well-being may differ in cross-cultural contexts (41). The HeadStart Learning Programme evaluation team are interested in assessing the effectiveness of these interventions, as well as using project data to investigate broader issues related to adolescents' mental health and well-being. The evaluation consists of survey and interview data collection, gathered annually over a 5-year period.

### Participants

The sample was drawn from the first annual wave of qualitative data collection in the HeadStart Learning Programme, the interviews were conducted in 2017 and comprised interviews with 82 young people. Prior to taking part in the research, school staff were asked by the evaluation team to invite young people to take part in the qualitative evaluation if they had either already received support from the HeadStart Learning Programme or if they may do so in future. Such support could consist of targeted, universal+, or universal interventions. Accordingly, young people were considered “at risk” if they were experiencing emergent emotional difficulties (e.g., anger, worries and fears, lack of confidence, self-harm) in the context of challenging home, peer group and/or school lives that were sufficient to warrant (or considered likely to warrant in the future) intervention as part of the HeadStart Learning Programme.

Of the 82 interviews, 11 participants were excluded from the current study because of their age (9–10), given the intended focus on adolescence. The transcripts of the remaining 71 interviews with 11–12-year olds were reviewed to determine if the young person discussed personal experience of emotional distress (sadness, anger, worries, anxiety etc.) and perceived cause for said distress; those who discussed both were included in the study (*N* = 32). There were instances where young people discussed what might be perceived by others as distressing scenarios, however, they did not report feelings of distress. Likewise, some young people discussed feelings of distress but did not reflect on cause. As the current study focuses on perceived cause for emotional distress from the perspective of young people, those who did not discuss emotional distress *and* cause could not be included. The excluded participants were checked and verified by the second author.

The final sample consisted of 32 young people, 17 males (53.1%) and 15 females (46.9%). Their ages ranged from 11.08 to 12.09 years old [mean (*M*) = 12, standard deviation (*SD*) = 0.31]. See Table 1 for further details.

**Table 1:** A summary of the demographic data for the young people included in the study.

**Demographic variable**	
Male	17 (53.1%)
Female	15 (46.9%)
Age range	11.08–12.09 [(*M*) = 12, (*SD*) = 0.31]
White British	22 (68.75%)
Mixed ethnic background	4 (12.5%)
Asian or Asian British	2 (6.25%)
Any other White background	3 (9.35%)
Black or Black British: African	1 (3.1%)

The qualitative strand of the national evaluation of the HeadStart Learning Programme received ethical approval from University College London (ID Number: 7963/002). Young people were given the opportunity to participate in the interviews, which they were free to accept or decline. Researchers conducting the interviews had up-to-date Disclosure and Barring Service checks and received safeguarding training from their host institution. Before young people who decided to take part were interviewed, they and their parents were asked to read a participant information sheet detailing the study. Thereafter, informed consent was sought by parents/carer(s), and assent to take part was sought from the participants before the interviews began. Interviewees were made aware that the information they provided would remain confidential, unless they disclosed something that indicated harm to themselves or others. During the write-up phase, all identifying information was anonymised; interviewees were aware this would happen.

### Data Collection

Data were generated through semi-structured interviews, ranging from ~15 to 60 min in length (*M* = 38.02, *SD* = 9.85), which were guided by an interview schedule developed as part of the evaluation of the HeadStart Learning Programme. The schedule was developed through internal review by the research team and “young research advisors” at Common Room—a consultancy and advocacy organization that supports the views of young people in research and policy.

The interview questions asked about young people's experiences of coping with problems and difficulties in their daily lives, including questions about what coping strategies they employed and their opinions on different sources of formal [e.g., from the HeadStart Learning Programme or other professionals] and informal (e.g., from family and friends) support that they had received. The young people were asked about this in various contexts, such as school-life, home-life, family, friendships, and feelings and emotions. The semi-structured nature of the interview schedule meant that the interviewer used these broad questions as a tool to guide the conversation with participants, using follow-up probes to elicit more detail as necessary. The interviews took place between the young people and members of the research team on a one-to-one basis in a private room at the young people's schools.

The focus of the interviews was to support the evaluation of the HeadStart Learning Programme, and, therefore, the focus was not directly related to adolescents' perceived cause(s) for emotional distress. However, when the adolescents were asked about their experiences of coping in difficult times, they also often talked about the perceived causes, either spontaneously or as a result of a prompt by the interviewer seeking to elicit further detail for clarification. Exploring young people's causal perceptions of emotional distress and help-seeking behavior also has implications relevant to the evaluation of the HeadStart Learning Programme and supporting young people's emotional well-being in general. Overall, the lack of direct focus on perceived cause for emotional distress has several benefits for the present study. The questions focus on several aspects relating to the maintenance of well-being and factors that contribute to sub-optimal functioning, thus, providing the opportunity to look at perceived cause(s) in the wider context of the participants' lives. Furthermore, understanding cause requires complex cognitive processes (30, 36) and, as they are still developing cognitively (37), young adolescents may find it difficult to consider what causes their emotional distress (29, 42). It may be more beneficial, therefore, for young people to engage in a wider conversation about their well-being where etiological beliefs can present themselves more instinctively without the pressure to directly reflect on cause.

### Data Analysis

Whilst cause for emotional distress is complex and multifaceted, this research sought to discover if meaningful categories of causal perceptions existed within a non-clinical sample. Ideal type analysis is the most appropriate form of analysis to achieve this, as it provides a systematic methodology for developing a typology from qualitative data. The construction of ideal types also allows for comparative analysis between and within the cluster of cases (40). This provides an opportunity to present clear distinctions between overarching cause(s) whilst delineating the nuance between cases in the assigned categories.

The concept of ideal types was first presented by Weber (43) to describe what is essentially an amalgamation of notable attributes from similar cases brought together to reflect how a particular phenomenon is understood (44). An ideal type is not the notion of something being perceived as *perfect* (45), but rather an explanatory schema used to understand the “infinite causal web” of social phenomena (40). The word “ideal” therefore describes “something that only exists in the mind” [47, p. 217], and is useful when exploring how adolescents perceive the cause(s) of their personal emotional distress. Drawing primarily on Stapley et al.'s (46) seven steps for ideal type data analysis the following procedures were observed.

#### Step 1: Becoming Familiarized With the Data Set

The first author (AON) had access to the transcripts and audio files prior to the development of the study and was already acquainted with the data. To ensure familiarity, AON re-read the transcripts and re-listened to the audio files, noting initial ideas and observations. At this stage, AON also took care to reflect on potential biases that could influence the interpretation of the data, such as knowledge of interviews from future timepoints. To ensure possible biases were kept in check, multiple co-researchers were involved throughout the analysis process to ensure findings were grounded in the data (46).

#### Step 2: Writing the Case Reconstructions

A summary (case reconstruction) of each of the 32 interviews was created, focusing on instances where emotional distress and perceived cause for emotional distress were discussed. The summaries were shared with a co-author, who was also highly familiar with the content of the interview transcripts, for feedback—changes were made to the case reconstructions as appropriate, these included adding more detail where necessary.

#### Step 3: Constructing the Ideal Types

Each case was systematically compared and contrasted with the other case reconstructions to identify patterns throughout the data. This was completed initially by AON and then again with a team comprising of the first author and two additional co-researchers. After the initial ideas were discussed, AON grouped similar cases into types to construct the ideal types, these were then shared with the team for feedback, and edits were made as appropriate.

#### Step 4: Identifying the Optimal Case

A case from each cluster was chosen that best illustrated the ideal type; this case would then act as an orientation point for comparison of other cases within that type.

#### Step 5: Forming the Ideal Type Descriptions

A detailed description of each ideal type was constructed, with the optimal case for each type in mind. As it is not necessary for every feature of every individual case to be represented in the ideal type description, the cases were represented by the description to varying degrees (47).

#### Step 6: Credibility Checks

In line with other research (44, 45, 48, 49), credibility checks were carried out to make certain that the ideal types and descriptions clearly reflected the data, and that each case was categorized under the most suitable type. To ensure this process was rigorous, it involved the input of three co-authors, including the first author and an independent co-judge. Initially, AON grouped the cases under the most appropriate ideal type by reading each case summary and assigning it to the type that best reflected the content, forming clusters. To verify that every case was placed accurately, ES also independently placed each case under the most befitting type. The clusters formed by the respective authors were then compared, and, in instances where cases were not placed under the same type, they were discussed until the appropriate placement was agreed between AON and ES. Initial consistency between the placements was <100%, therefore, discussions and feedback from the sorting processes fed into the redrafting of the ideal type descriptions to ensure absolute clarity. Finally, an independent co-judge was given the clusters, on separate occasions, without being told which type they belonged to. The co-judge was asked to assign each cluster to a type using the ideal type descriptions, this was done with 100% accuracy.

#### Step 7: Making Comparisons

Once homogeneity of the types was successfully delineated, the heterogeneity within the clusters was examined in order to capture the extent of the variability and nuance within the types.

## Results

### Demographic Data

Table 2 outlines the demographic data for each of the five ideal types. As indicated, “Others” is the most ethnically diverse category. “Perceived lack of control” hosts the youngest participant, whereas the oldest participants can be found in “Unfair treatment” and “Others.”

**Table 2:** A summary of the demographic data for the young people included in the study, organized by ideal type.

	**Ideal type (total** ***N*** **of cases)**				
	**Perceived lack of control (*N* = 4)**	**Unfair treatment(*N* = 6)**	**Others (*N* = 12)**	**Concern for self and others(*N* = 4)**	**Self as cause (*N* = 6)**
**Demographic variable**					
Male	3 (75%)	3 (50%)	5 (42%)	3 (75%)	3 (50%)
Female	1 (25%)	3 (50%)	7 (58%)	1 (25%)	3 (50%)
Youngest participant years & months	11.8	11.10	12	12.1	11.11
Oldest participant years & months	12.4	12.9	12.9	12.7	12.5
White British	2 (50%)	5 (83%)	7 (58%)	3 (75%)	5 (83%)
Mixed ethnic background	1 (25%)		2 (17%)	1 (25%)	
Asian or Asian British	1 (25%)				1 (17%)
Any other White background		1(17%)	2 (17%)		
Black or Black British: African			1 (8%)		

### Ideal Types

It is important to note that the young people interviewed could be placed into a number of these categories, as causal perceptions for emotional distress are complex and varied. However, participants have been placed into the category which best illustrates the over-arching focus of their causal perceptions. The five types are presented below alongside ideal type descriptions, the optimal case from each group, and a summary of other cases within the cluster.

#### Ideal Type 1: Perceived Lack of Control

Four cases were represented, to varying degrees, by perceived lack of control.

##### Ideal Type Description

Young people in this group appear to be particularly affected by things they perceive to be out of their control. Being controlled by others, not being able to control who stays in their life, and feeling like they do not have control over how they feel/act are some of the instances that may cause emotional distress within this group. The young people in this group perceive their lack of control as something that affects their lives, they get distressed when things go wrong and may blame external causes for their problems—e.g., “it's the game's fault that I died,” “mum's fault my dad isn't around,” “his fault that I punched him” etc.

##### Optimal Case

The optimal case for this ideal type was Tom. Tom's emotional distress appears to manifest, in part, as a result of his struggle to define his own identity in the wake of his mother's “puppeteering.” For instance, Tom feels that he is “*a completely different person*” at his mum's; he describes it as a prison because he feels like he cannot be himself. Tom perceives his mother to be controlling and feels as though she tries to make him do things against his will, such as making him clean his bedroom. Tom wants to live with his father because he believes that it will give him the freedom that he craves and does not have while living with his mother. However, Tom also fears losing his father:

*Like, like, my mum and my dad just broke up as I was born… and… my dad told me that he, he could've been like most dads in the world… and he coulda just left and not wanted to know me, but he looked back… and he says I never want to regret that cis-, decision […] but your mum's pulling me away from you… and I don't really want to be away from you, but if it happens you're just gonna have to learn to deal with it… and it gets me upset*.

Tom also explains that his dad hit him once which made him cry. However, it was not being hit that made him upset but rather the shock and resignation that the dynamic of their relationship had changed: “*I was, like, oh, okay, don't, I can't mess with, around with daddy anymore*.” Ultimately, Tom appears to be affected by the realization that he has little say in how his father features in his life; he cannot choose to live with him, he cannot stop him from leaving, and he just has to accept when dynamics in their relationship change. However, not all of Tom's difficulties around perceived lack of control are linked to his family dynamics.

Tom states that he is physically and mentally unwell and believes he just has to “*get on with it*,” implying that he does not have control over his physical and mental well-being. He also expresses that he does not feel that he has explicit control over what emotion he will feel and that his brain chooses what he feels, thus, emotional distress is caused by perceived lack of control:

*obviously, no one wants to be sad, no one wants to be angry, so in that situation [my brain is] not just gonna pick me being angry. It's just gonna pick me being s-, it's gonna pick me being sad, and I'll just end up being that one*.

There are particular instances, nonetheless, when Tom describes explicit control over how he presents himself, such as masking sadness when around peers. However, these reactions appear to be his way of protecting himself from ridicule and seem to be prompted by previous bullying experiences, which again he sees as outside of his control. This is evidenced by his confidence that certain peers will react negatively to emotional displays, “*but if I cry I'm just gonna leave them and they'll be, like, oh, he's a wimp*,” indicating that Tom knows he lacks control over the outcome of these instances.

##### Summary of the Other Cases Within this Type

The other young people in this cluster also present perceived lack of control as the overarching cause for their emotional distress. Some of the cases are similar to Tom; Jane, for instance, also struggles with a controlling parent and finds the lack of control difficult to manage. When speaking about her father, Jane states: “*he done like things that I didn't like, like controlling, stuff like that so. That actually made my confidence a bit lower, ‘cause like, there’s someone control you, it just don't feel right.”* Unlike Tom, Steve's difficulties with perceived lack of control are centered around school rather than home, such as not having the teacher he wants. Steve also finds gaming to be a trigger for emotional distress; when asked about the “*rage*” he associates with gaming, he explains: “*I get angry. Firstly, I start swearing at my phone when I'm playing this game. Then I start hitting the sofa*.” In Charlie's case, losing video games also makes him angry and he too blames the game. For Charlie, things going wrong also makes him stressed—he says that people call him a perfectionist, indicating a need for control. However, like Tom, not being able to control who stays in his life is also distressing for Charlie.

#### Ideal type 2: Unfair Treatment

Six cases were represented, to varying degrees, by unfair treatment.

##### Ideal Type Description

Young people in this group feel as though they are treated differently from their counterparts and perceive this treatment to be unfair and, therefore, distressing. They may also feel that others do not understand them which causes, in their opinion, people to treat them differently and less favorably to others. For instance, the young people in this group tend to feel that teachers shout at them unfairly and do not listen to them.

##### Optimal Case

The optimal case for this ideal type was Anna. Anna feels that she is often on the wrong side of favoritism and finds this unfair treatment distressing. For instance, she loves dancing, but feels that her dance teacher gives other students more, “*[s]o he focuses on him more than me. So then I get upset*.” Anna explains that favoritism makes her cry, indicating that it makes her feel “*[a]nger and tired, like, anger and tired*.” In Anna's view, teachers tend to treat her less favorably and often do not believe her when she confides in them. She feels as though she is unjustly singled out by certain teachers, and this injustice makes her angry: “*I don't care who I work with, it's just that, like, people, people like single me out. That's what I hate*.” This perceived unfairness, above all else, appears to trigger emotional distress. Anna also believes that she was disliked and bullied at her old school because of her nationality: “*[b]ecause I'm [*states nationality*]. Like, they didn't understand what being different was*.” She feels that she was treated differently as a result which led to events that caused emotional distress, thus, she perceives unfairness to be the starting point of her distress.

##### Summary of the Other Cases Within this Type

Like Anna, five other young people perceived unfair treatment to be the most prominent cause of emotional distress. Chloe feels like she is treated differently by peers who treat her like a “*little kid*” and teachers who are unfair to her. When Cohan discusses things that make him angry, he states: “*[g]etting yelled at, getting made fun of, people, teachers, when they have a go at me for not doing something right… getting treated with less respect than some of the other pupils*.” Similarly, Aaron perceives receiving less respect and unfair treatment from teachers as causes of emotional distress, and Danny indicates that he also gets “wound up” a lot by unfair treatment.

Unlike Anna, Tilly focuses more on inequalities at home as a cause for emotional distress rather than at school. Tilly feels it is unfair that she has to live with her dad because he lied to social services about her mum and he appears to neglect her. Additionally, Tilly feels that her dad gives her brother more attention and that he does not really take notice of her, her brother also used to hit her and she was affected by the fact that her dad did not believe her. Like Tilly, Cohan is also affected by family issues, he feels helpless regarding family problems and appears to feel that it is unfair that he cannot do anything to help: “*Nothing, like, (sniffs) just can't, like, stop my mum from feeling pain, I can't s-, help my dad, too much… cause I'm not legal age to work*.”

#### Ideal type 3: Others, Their Actions, and Judgements as the Catalyst

Twelve cases were represented, to varying degrees, by others, their actions and judgements being the catalyst for emotional distress.

##### Ideal Type Description

Young people in this group tend to attribute their experience of emotional distress to the actions or the perceived judgement of others. For young people who fall under ideal types 1 and 2, the actions of other people indirectly influence cause as they tend to focus on injustice or the lack of control rather than the action itself. In this group, however, the explicit action(s) or perceived thoughts of others appear to act as a catalyst for emotional distress. Distress within this group can also be caused by the thought of how someone *might* act toward or judge them.

##### Optimal Case

The optimal case for this ideal type was Billy. Billy finds the classroom frustrating and he perceives the actions of his peers to be a cause of emotional distress: “*Kicking on the desk, little whispers of voices [Int: how does it make you feel?] Really angry, like angry, angry. And they make me feel… scared, in a weird way*.” It made Billy “*really, really sad*” that a friend stopped talking to him because he uses headphones to cope with the noise—further indication that actions and perceived judgements cause him distress. Billy explains that he can often become distressed in school but suggests that he is not always taken seriously by members of staff. For instance, he used to be allowed to leave class when he felt distressed but then this was revoked by teachers, which left him feeling “confused” and “sad.” Thus, Billy perceives the teacher's actions to be a cause of emotional distress to the extent that he experienced a panic attack as a result.

Billy believes that his panic attacks started because of being bullied and threatened by his peers. At one point, his panic attacks lessened, however, a further incident of threatening behavior from peers brought them on again. It is not just the actions of his peers, however that Billy perceives as a cause for emotional distress, he is also affected by their (perceived) judgements. For example, when people call him short and cute it really gets him down as he does not appear to view these as desirable qualities. He also feels that if he were popular his life would be better and that he would not experience emotional distress to the same degree: “*I could have had this big chance at being the popular one, and I wouldn't have any of these problems if I did. I have my regrets*.” This further indicates that Billy perceives the judgements and actions of others to be a cause for emotional distress.

##### Summary of the Other Cases Within this Type

Eleven other young people were assigned to this type because they also perceive the actions and judgements of others to be the overarching cause of their emotional distress. Like Billy, Rory also finds that people making noise in school makes him feel stressed. For Rory, however, he is irritated by relentless swearing. Susie, on the other hand, appears to be very affected by what other people think of her and how they might react if she gets something wrong: “*that's what I worry about like, getting something wrong*.”

For some young people in this group, it is the actions of bullies and peers who are rude/pick on them that appears be the focus of causal perceptions. For instance, when Scott was asked what makes him upset, he stated: “*Like people cussing me. People, like, taking the mick and stuff like that*.” Similarly, Flynn and Lilly have been affected by name calling and disrespect from other students. For Cathy, however, it is people starting and spreading rumors that makes her feel “*upset*” and for Samantha, it is others leaving her out that causes emotional distress: “*if they just, like, leave me out or something I'd get sad*.”

Young people in this group also indicated that they found the actions of fathers/stepfathers to be a cause for their emotional distress. For instance, Rory also expresses how he found his dad's behavior to be distressing as he is unreliable: “*All I can say is, is that he's also a let-down, that's all I can say*.” Likewise, Corry also has issues with his dad which appear to cause distress. As for Kathy, arguments at home cause her difficulties: “*I don't really cope with it*.” She says that she can get very angry during the arguments and that her stepdad makes the anger worse. Kathy further indicates that her biological father used to abuse her mum and sisters, he keeps turning up in their life uninvited. Maggie also repeatedly reports the actions of her stepdad as a cause of emotional distress. She indicates that her stepfather is aggressive and potentially has an alcohol problem: “*sometimes when he gets really angry and he's drunk beer during the day, he h- hits me. (sniffs)*.” She says she sometimes gets “*really upset*” about it.

#### Ideal type 4: Concerns for Self and Others

Four cases were represented, to varying degrees, by concern for self and others.

##### Ideal Type Description

The young people in this group indicate that concern for others who are experiencing, or who may experience, difficulties causes them distress—this may be their friends, family, or stories they hear on the news. They are also concerned with their own welfare and the part they might play in affecting other people, which makes them eager not to cause disappointment or burden others with their problems.

##### Optimal Case

The optimal case for this ideal type was Paula. For Paula, concern for the welfare of others is a significant cause of emotional distress. For instance, she claims, “*I don't like to see my friends upset and that, like, when they get upset, I get upset as well*.” Concern as cause is also true in relation to the well-being of her family. For instance, when Paula discusses how she felt hearing that her sister had been attacked she states:

*I found that hard, but, but at school people don't take things seriously and, like, they go ha-ha I've just been [unclear 00:22:19]. And found that hard as well and it's, like, it's not something to joke about. It's like it has happened to people. but. it is hard like to cope with things*.

It is clear that Paula perceives other people experiencing difficulties and people being mean to others to be a direct cause of emotional distress for her, as she further states:

*[I]t's like if people, like, are being mean to people I find it, like, really upsetting. Like if I watch something I find that upsetting as well, like if it's, like, someone getting bullied I find it upsetting. And then, like, I watched this programme […] I sat on the settee and I just cried. […] It's like the other week there was this little girl and she couldn't walk or nothing and I just found it so upsetting, she had to live on the couch, [.] she couldn't move and it was upsetting*.

Not all of Paula's emotional distress in relation to concern is centered on other people. She has also felt upset because her dog had to go to hospital without her. Paula was also eager not to burden her parents with her problems when they were supporting her sister. This indicates that she did not want to take attention from her sister and was worried how her actions may have impacted her sister's welfare.

##### Summary of the Other Cases Within this Type

Three other participants were allocated this type as they indicated that concern for themselves and others was an overarching cause for their emotional distress. Like Paula, Matt's emotional distress is centered on concerns for the welfare of his family. However, Matt's emotional distress appears to be the result of concerns regarding how he might disappoint his family: “*well, I'm, I'm really worried about French because, honestly, […] Er. how my parents will feel, especially my granddad […] I'm worried that I'll disappoint them if I get bad scores on it*.” Ciaran's perception of cause is also centered around concerns for himself and others. When asked what makes him worried, he states: “*if it's that I hear something that, if I hear something about maybe my parents or something that might happen to someone or what's going on now in the news*.” Paul, on the other hand, states that getting in to trouble makes him sad because no one will play with him and he fears telling his parents about getting in trouble because of their potential reaction. Thus, it is concern for himself that prompts the emotional distress. However, Paul has also hurt people in the past and concern for how he has affected them has caused emotional distress, as he states: “*uh… hurting other people's feelings makes me upset*.”

#### Ideal type 5: Self as Cause

Six cases were represented, to varying degrees, by self as cause.

##### Ideal Type Description

Young people in this group feel that they play causal role in the manifestation of their emotional distress. They will hold themselves accountable for the way in which they react to the behavior of others as cause for their distress rather than the questionable behavior. Some may feel that their disposition is the reason they experience uncomfortable emotions, for instance, they may accuse themselves as having a short fuse or being implosive. The problems that they face are, more often than not, not seen as a cause for emotional distress but the way in which they deal with the issue thereafter is. There is also an element of emotional management as a factor in perceived cause for emotional distress; the experience of one emotion appears to roll into a multitude of emotions like a domino effect. Thus, the young person feels that the way in which they managed the initial emotion plays a part in the subsequent distress.

##### Optimal Case

The optimal case for self as cause was Sarah. There are a number of ways in which Sarah holds herself accountable for her experience of emotional distress. For instance, Sarah acknowledges that she may overreact in situations she finds frustrating which will inevitably lead to distress: “*When sometimes like annoys me or, like irri-, irritates m, like I think I have a really short fuse, so like I've literally got about five […] seconds and I will explode*.” She explains she has been learning how to have more patience so “*it'll take more stuff to like…explode*,” indicating that she views emotional distress to be linked to her capacity to mediate her response. When discussing her “*short fuse*,” she states retrospectively, “*if somebody said something, like, nasty or something like that, straight away I'd snap back at ‘em*,” she explains that she would get really angry if she did not say anything back. Thus, the suggestion that her anger comes because of her inaction rather than what was said implies a view of self as cause for emotional distress.

Sarah also perceives personal characteristics, such as being disorganized as a cause for emotional distress. Further, she explains that she used to “*getting real, like…panicked in school*” because she felt like she did not fit in, again suggesting that she views personal attributes to be responsible for emotional distress. Sarah used to find it difficult to discuss her problems with others, “*I've always been really independent but like, I sort of kept it to myself and that*.” She now speaks to her mum about her concerns and she sees this as a better alternative to keeping concerns inside and worrying all day, indicating that keeping things inside was the cause of worry rather than the actual concerns.

##### Summary of the Other Cases Within this Type

Five other cases were represented by self as cause. Ryan for instance, appears to feel that he needs to control how he might feel in reaction to his parent's behavior: “*it's like the kind of thing you can get angry about […] or you feel you don't want to get angry about […] For example my dad drinks a lot of alcohol and I think it's bad for him*.” Similarly, Riley feels that the way in which they manage their problems is causal: “*I don't like when things are stuck inside me, like, it makes me want to feel like I need to cry […] it's just like, building up inside me and just can't deal with it anymore, so I just tell someone*.” Holding in problems is seen as the cause of emotional distress rather than the actual problem, thus, the self is seen as causal, Amy also feels that holding on to problems causes emotional distress. For Luke, however, he feels emotions create emotional distress, like a domino effect:

*Like nervous, because when I'm nervous I get stressed and start asking myself questions that sometimes I can't answer. Um… when I'm angry and stressed… um… so… when I'm nervous, which makes me stress, is sometimes I bite my nails, um… and then that stresses me out*.

Luke claim*s* he asks himself these questions out of stress that make him feel more stressed, further indicating that he perceives how he manages emotions to be causal. Tommy, on the other hand, feels that he is implosive: “*I feel angry sometimes but like I don't show it. I'm very implosive*.” He further states that he tries to keep his feelings “*locked away*” but says sometimes they can “*leak*.” This indicates that Tommy feels he plays a role in the cause of his emotional distress insofar as he failed to keep his emotions from “leaking”.

## Discussion

The aim of this study was to develop a typology of adolescents' perceived cause(s) of emotional distress. We sought to investigate if meaningful categories of causal perception existed within a non-clinical sample, as has previously been found in clinical samples using thematic analytical approaches (29). Using ideal type analysis, we identified five distinct categories: (1) *perceived lack of control*; (2) *unfair treatment;* (*3*) *others, their actions and judgements as the catalyst;* (4) *concerns for self and others; and*, (5) *self as cause*.

It is important to note that the ideal types arguably correspond to very general experiences of young adolescents. However, adolescents' emotional difficulties are often dismissed as “typical” and their individual needs tend to be overlooked (4, 50). Our participants indicated that they find certain experiences emotionally distressing, suggesting that some young people may require more help navigating these experiences than others. Further, whilst it is possible to hold several causal attributions, the young people in this study were placed into the category that best represented the overarching cause of their emotional distress; the types are broad and nuanced to reflect the complexity of the causal attributions.

The first type, *perceived lack of control*, indicates that some young people may attribute their experience of distress to a lack of perceived agency in their lives, as well as their behavior and emotions. Perceived control is an important factor for optimal psychological functioning (51, 52), therefore one's locus of control—the perceived degree to circumstances are caused by internal or external factors—has important implications for well-being. External locus of control has been linked with negative outcomes, including psychosis and depression (52–54). The participants in this category appear to be describing an external locus of control. Thus, our findings support conclusions that a lack of perceived control contributes sub-optimal well-being [e.g., (55, 56)]. The notion of a lack of control being potentially distressing to adolescents is not surprising, given that striving for independence is a quintessential feature of this epoch (57). However, it has been argued that a sense of primary control (control over the external environment) and secondary control (adjustment to and acceptance of situations) develop at different rates, with secondary control taking longer develop (58). Therefore, lack of control may be a more significant cause of emotional distress in early adolescence, and it is possible that the young people in this category have yet to develop secondary control, explaining why it has a distinct pattern.

Lack of control within this type, however, was not limited to external stimuli, but also a lack of perceived control over thoughts, emotions, and behavior. Having control over thoughts and impulses accounts for 2 of the 4 major domains of self-control (59). It has been argued that self-control facilitates coping (e.g., activities used to manage difficulties), as it allows individuals to modify the impact of undesirable emotions (60). Indeed, Finkenauer and colleagues (60) found that lower self-reported levels of self-control were strongly related to emotional and behavioral issues in early adolescence. Effective coping strategies are known to enhance resilience in the face of adversity, owing to the ways in which they help individuals manage their difficulties (61). Therefore, these particular overarching causal beliefs may exist due to the way in which lack of perceived agency impacts normative coping strategies.

The second type, *unfair treatment*, highlights that, for some young people, what they interpret as unfair treatment may become the overarching cause of their emotional distress. This supports both recent findings that perceived injustice in an educational context may have a significant impact on the lives of adolescents by potentially influencing dishonesty (62), and earlier studies showing that unfair treatment has implications for emotional states and behaviors (63, 64). Research has observed that there are variations in sensitivity to unjust treatment (62, 65), which may explain why this type is an overarching cause for some and not others. These findings, therefore, highlight that young people interpret events differently, but also demonstrate that young people may experience unfair treatment in comparison to their peers/sibling etc. The *unfair treatment* type also illustrates that those who perceived unfair treatment to be the overarching cause of their emotional distress often feel that they are being mistreated by “significant others” (teachers/parents/peers), sometimes believing this is because they are “different.” Significant others are important support systems (21), thus young people who experience unfair treatment may not feel they have access to the same sources of support as their counterparts.

Ideal type 3, *Others, their actions and judgements as the catalyst*, represented the largest cluster in the study. This is perhaps unsurprising given that adolescents tend to be more sensitive to how others view them (57, 66). This category indicated, however, that it is not only the thoughts and actions of others that young people perceive as a cause of distress, but also the imagined thoughts and actions of others. Moreover, most of the individuals in this group were previous or current victims of bullying, indicating that those who have been victims of bullying may be more sensitive to the thoughts and actions of others. Indeed, globally, bullying victimization has been found to be prevalent amongst adolescents (67) and, there is little doubt among scholars that bullying is an adverse experience that will likely negatively impact well-being (68).

The fourth category, *concern for self and others*, indicates that some young people feel that concern is the overarching cause for their emotional distress. Research demonstrates that young people who have an ill family member experience distress as a result of a number of factors, one of which is concern about the progression of the illness which may affect the lives of those they care about [e.g., (69–71)]. The present research supports these findings, and adds that worries about what *might* happen to a loved one, even if they are not currently facing difficulties, can cause distress. The findings from this study indicate that some of this concern may be perpetuated by negative stories in the news or on social media. Indeed, findings from earlier research by Riddle and colleagues (72), indicates that young children may be frightened by negative news content, and are able to vividly recall disturbing news stories. The present research adds that adolescents may too be emotionally affected by negative news content.

The final cluster, *self as cause*, supports earlier findings from Midgley and colleagues (29), which indicated that young people may feel that something inside is to blame for causing their depression, resulting in negative attribution styles. The present research illustrates that self-blame transcends a clinical diagnosis and can also be observed as a perceived cause for emotional distress in “at risk” adolescent groups. These combined findings support conclusions drawn by Yeo (73) that amplify the need to change discourse around well-being to avoid focusing on “deficiencies” and messages about “keeping positive,” which infer it is the responsibility of young people to control how they feel. Young people may find attributing cause for emotional difficulties therapeutic (29). However, if they blame themselves for their difficulties it may have negative consequences for their self-perceptions, which are beginning to crystallize in adolescence (57). This amplifies the need to identify and resolve potentially negative perceptions regarding internal cause(s) of emotional difficulties before they are reinforced and form part of the young person's identity. Further, adolescents' emotional difficulties are often dismissed by influential groups of people, such as teachers and parents, as “typical teenage mood swings” and, thus their individual needs are often overlooked (4, 68). If significant others, salient to identity development, consistently reinforce a message of “typical teenage hormones” as an explanation for emotional distress, it is reasonable to theorize that adolescents may internalize cause, believing that their difficulties are innate.

## Implications

Our study indicates that adolescents attribute numerous factors to the cause of emotional distress and that young people may be affected by these factors to varying degrees. Indeed, based on the types of perceived cause, there may be a number of practical solutions that preventive programmes could develop in order to reduce emotional distress. For instance, our study suggests that empowering young people to speak out when they feel they are being treated unfairly, as well as encouraging people in their lives to actively listen and effectively respond to these concerns, may have positive implications for well-being. Similarly, involving young people in decisions that impact their lives, and exploring ways to ensure their voices are amplified, may help reduce emotional distress. In order to support this, it is likely necessary to work with individuals in young people's lives to (a) ensure they treat young people fairly and explain the reasoning behind their actions (b) help them create meaningful ways to involve young people in decision making. Further, our findings suggest that providing resources and support to improve young people's digital literacy, with the aim of helping them navigate emotive media and diminish its impact, may work to reduce potential emotional distress.

In addition, our findings raise possible implications for help-seeking behavior. The results demonstrate that the ways in which people interact with young people, the implications of these interactions, and the degree to which young people are concerned for how they impact others may engender emotional distress to varying degrees. Therefore, to some extent, all of the types have inextricable links to the people adolescents interact with in different contexts (school, home, media etc.). These links introduce likely implications for help-seeking, particularly as significant others, including peers and teachers, are typically the key to helping struggling young people identify and access appropriate support for their difficulties [e.g., (74)], and are an important support system in their own right. Our study suggests that young people may feel ignored by or distrusting of significant others, thus removing important support systems (23). Consequently, investing in additional training to ensure those who are important in the lives of adolescents are aware of how their words and actions may be perceived, as well as how to manage their impact, may help foster a supportive environment that is conducive to help-seeking.

However, our study also indicates that some young people find the *possible* impact they have on others distressing, and may, therefore, not want to share their problems to avoid additional distress. Indeed, according to Yeo (73) some young people feel that their issues are too burdensome for others, which can prevent them from disclosing their experiences. Similarly, we found that some young people, particularly those who have been bullied, are affected by the *perceived* thoughts of others and, thus, may not seek help as a form of self-preservation. Consequently, developing ways for young people to access support anonymously may be beneficial for some groups. This could include having a dedicated area on a school website with helpful resources they can easily and privately access. It may also be worthwhile working with students to co-develop resources and alternative help-seeking routes that complement a diverse range of help-seeking needs.

Our findings provide a foundation for research seeking to investigate the causal beliefs of emotional distress in relation to help-seeking in non-clinical groups. It is clear, however, that longitudinal research on perceived cause for emotional distress is required to better understand its development and, subsequently, to allow for timely administration of targeted interventions. Likewise, work is needed to map out the ways in which perceived cause may influence help-seeking to better support the needs of students and to create practical and diverse help-seeking solutions. Future research should, therefore, endeavor to identify whether a link between perceived cause(s) for emotional distress and help-seeking exists in non-clinical groups.

## Limitations

The results from the present study were generated through secondary data analysis, as the main focus of the interviews analyzed was to support the evaluation of the HeadStart Learning Programme. However, the interview questions did focus on several aspects relating to well-being and sources of support, thus, providing the opportunity to look at perceived cause(s) in the wider context of the participants' lives. Yet, the fact that perceived cause was not the sole focus of the interviews meant that the degree to which young people discussed causal perceptions in their interviews varied. Further, the sample in this study consisted only of young people who were identified by their teachers as currently receiving, or who may receive support from the HeadStart Learning Programme. Thus, the degree to which the results from this study are reflective of the causal perceptions of young people who experience elevated levels of emotional distress but are unknown to their teachers, or to the HeadStart Learning Programme is currently unknowable.

Further, as outlined by Stapley et al. (49) there are limitations to using ideal type analysis as a form of data analysis, including the reality that smaller details of participant interviews may be omitted in the process of analysis. This is because condensing large amounts of detailed interview data is necessary in the early stages of analysis to create brief case reconstructions that reflect the aim of the study. Although less detailed, the authors took care to ensure each transcript was summarized to accurately reflect the most salient features of the interviews, ensuring pertinent details were not lost.

## Conclusion

This study illustrates that distinct categories for perceived cause for emotional distress can be observed in non-clinical adolescent groups, a finding which may have important implications for help-seeking and overall well-being more generally. It highlights the need for future research to explore the possible links between causal beliefs and willingness to seek help. Such insight may prove beneficial to the development of timely and effective interventions that seek to improve adolescent well-being and prevent the onset of affective disorders.

## Data Availability Statement

The data analyzed in this study is subject to the following licenses/restrictions: The interviews were generated as part of a national evaluation of an early intervention programme and are held by the Anna Freud Centre. Requests to access these datasets should be directed to emily.stapley@annafreud.org.

## Ethics Statement

Ethical review and approval was required for the study on human participants in accordance with the local legislation and institutional requirements. Written informed consent to participate in the study was provided by the participants' legal guardian/next of kin.

## Author Contributions

AO'N, ES, and NH contributed to conception and design of the study. AO'N selected the participants and summarized the interviews and wrote the first and subsequent drafts of the manuscript. AO'N and ES performed the analysis, SS assisted with creating the initial categories for analysis. SS and HM checked and verified the analysis. All authors contributed to manuscript revision, read, and approved the submitted version.

## Conflict of Interest

The authors declare that the research was conducted in the absence of any commercial or financial relationships that could be construed as a potential conflict of interest.
